# Combinations of Freeze-Dried Amorphous Vardenafil Hydrochloride with Saccharides as a Way to Enhance Dissolution Rate and Permeability

**DOI:** 10.3390/ph14050453

**Published:** 2021-05-11

**Authors:** Gabriela Wiergowska, Dominika Ludowicz, Kamil Wdowiak, Andrzej Miklaszewski, Kornelia Lewandowska, Judyta Cielecka-Piontek

**Affiliations:** 1 Tarchomin Pharmaceutical Works “Polfa” S.A., A. Fleminga 2, 03-176 Warsaw, Poland; Gabriela.Wiergowska@polfa-tarchomin.com.pl; 2 Department of Pharmacognosy, Poznan University of Medical Sciences, Swiecickiego 4, 60-781 Poznan, Poland; dsiakowska@ump.edu.pl (D.L.); the3nigmav2@gmail.com (K.W.); 3 Institute of Materials Science and Engineering, Poznan University of Technology, M. Sklodowskiej-Curie 5, 60-965 Poznan, Poland; andrzej.miklaszewski@put.poznan.pl; 4 Institute of Molecular Physics, Polish Academy of Science, Smoluchowskiego 17, 60-179 Poznan, Poland; kornelia.lewandowska@ifmpan.poznan.pl

**Keywords:** vardenafil hydrochloride, amorphous dispersion, apparent solubility, permeability

## Abstract

To improve physicochemical properties of vardenafil hydrochloride (VAR), its amorphous form and combinations with excipients—hydroxypropyl methylcellulose (HPMC) and β-cyclodextrin (β-CD)—were prepared. The impact of the modification on physicochemical properties was estimated by comparing amorphous mixtures of VAR to their crystalline form. The amorphous form of VAR was obtained as a result of the freeze-drying process. Confirmation of the identity of the amorphous dispersion of VAR was obtained through the use of comprehensive analysis techniques—X-ray powder diffraction (PXRD) and differential scanning calorimetry (DSC), supported by FT-IR (Fourier-transform infrared spectroscopy) coupled with density functional theory (DFT) calculations. The amorphous mixtures of VAR increased its apparent solubility compared to the crystalline form. Moreover, a nearly 1.3-fold increase of amorphous VAR permeability through membranes simulating gastrointestinal epithelium as a consequence of the changes of apparent solubility (P_app crystalline VAR_ = 6.83 × 10^−6^ cm/s vs. P_app amorphous VAR_ = 8.75 × 10^−6^ cm/s) was observed, especially for its combinations with β-CD in the ratio of 1:5—more than 1.5-fold increase (P_app amorphous VAR_ = 8.75 × 10^−6^ cm/s vs. P_app amorphous VAR:β-CD 1:5_ = 13.43 × 10^−6^ cm/s). The stability of the amorphous VAR was confirmed for 7 months. The HPMC and β-CD are effective modifiers of its apparent solubility and permeation through membranes simulating gastrointestinal epithelium, suggesting a possibility of a stronger pharmacological effect.

## 1. Introduction

Vardenafil hydrochloride (VAR) is a selective phosphodiesterase V (PDE-5) inhibitor that exhibits vasodilatory activity [1,2]. By inhibiting the degradation of cyclic guanosine monophosphate (cGMP), it causes prolonged relaxation of a muscle, dilation of vessels, and increased blood flow in the penis, resulting in erection [3,4]. VAR has a comparable but slightly prolonged duration of pharmacological effect with regards to sildenafil—the first PDE-5 inhibitor used to treat erectile dysfunction. It is recommended to take VAR about one hour before intercourse, but patients can use it from 30 min to 2 h before sexual activity, as the medication requires sexual stimulation for effectiveness [5]. VAR is reported to be a very potent and highly selective inhibitor of PDE-5, which inhibits PDE-6 minimally as well. This selectivity prevents adverse effects, which sometimes occur with sildenafil, and ensures no VAR impact on visual acuity and color perception [6]. VAR is not only effective in the treatment of erectile dysfunction (ED), but it also has indications in premature ejaculation [7]. Some researchers present the effectiveness of VAR in pulmonary arterial hypertension [8]. Moreover, it has recently been reported that VAR might be beneficial in the treatment of cardiovascular diseases and has a protective effect on vascular endothelium and myocardial cells [9], which can be related to its antioxidant activity [10]. Furthermore, it has been suggested that VAR could be an interesting agent in the co-treatment of erectile dysfunction and osteoporosis in men due to its ability to improve osteoblastic bone formation along with inhibition of osteoclast formation [11]. In addition, it has been proposed that VAR can be considered a novel drug for cholestatic liver damage, thanks to its hepatoprotective activity, which could be associated with antioxidant and anti-inflammatory properties [12].

**Scheme 1:**
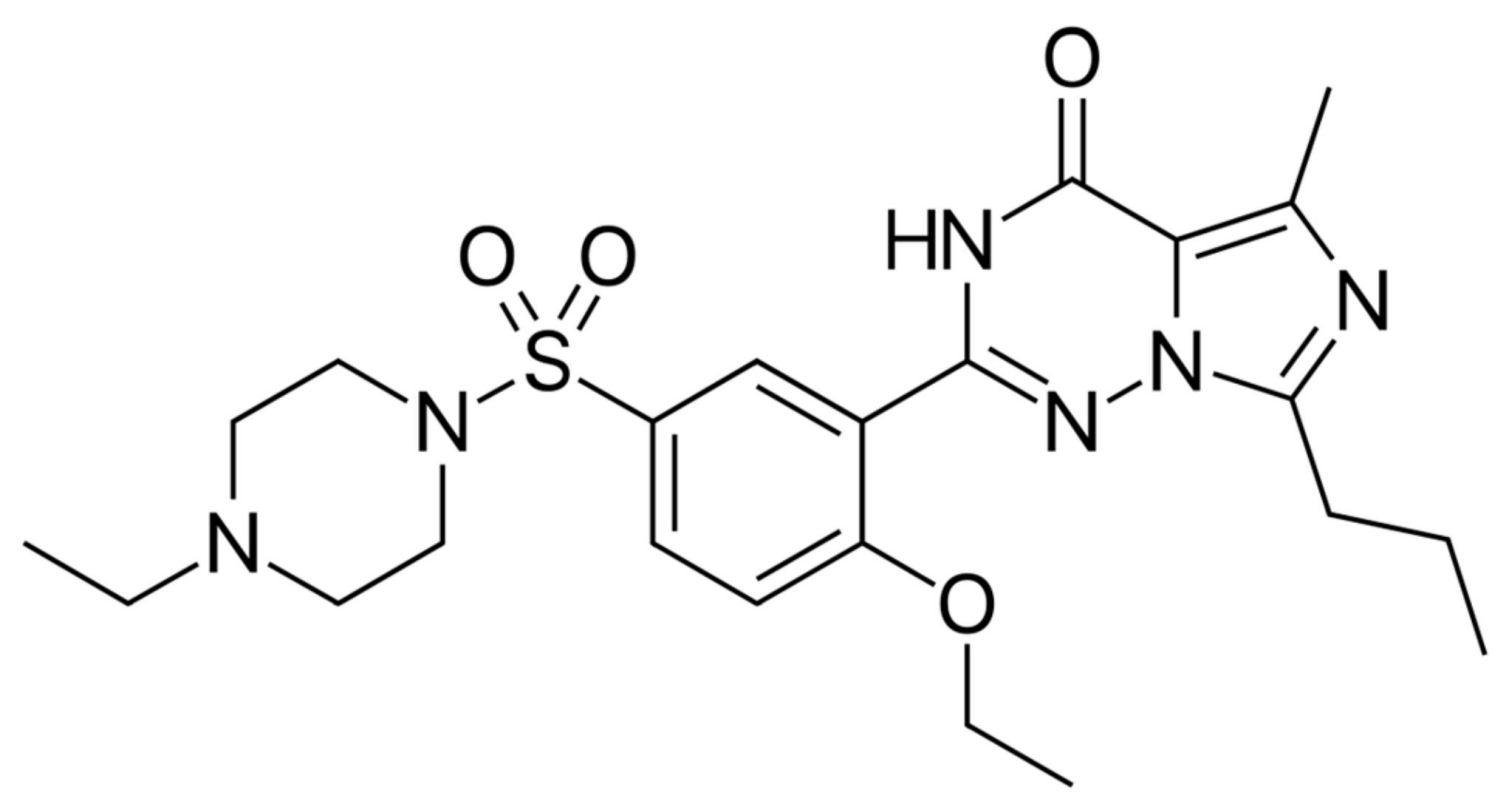
Vardenafil structure.

Vardenafil (Scheme 1) belongs to class II of the Biopharmaceutics Classification System (BCS) because it is classified as a poor soluble active pharmaceutical ingredient (API) with high permeability [13].

Its oral bioavailability is low—approximately 15%—due to low solubility (water solubility 3.5 mg/L) and extensive first-pass metabolism [5,14]. Various approaches are used to eliminate these limitations. The first is obtaining salts with better solubility. Such procedures are already used in the development of formulations, where vardenafil is used in the form of hydrochloride e.g., Levitra^®^ [15]. Another viable approach, suggested by other researchers, is changing the site of absorption of VAR via the development of orally disintegrating formulations or via the inhalation route of administration [16,17]. In this way, it is possible to prevent hepatic metabolism and therefore to achieve better bioavailability [18]. In addition to changing the chemical structure of API and selecting and optimizing appropriate pharmaceutical dosage forms, API’s physical structure modifications are also used [19,20]. Obtaining subsequent polymorphic forms of API often enables solubility to be modified [21] while preparing amorphous API dispersions, accelerating their dissolution [22]. Acceleration of API solubility induces faster absorption through biological membranes and ultimately stimulates the achievement of a pharmacological effect.

Currently, amorphization is considered one of the best methods to deliver insoluble drugs, especially those belonging to the II and IV BCS groups [23,24]. Numerous cases of increased solubility of API after obtaining amorphous dispersion have been described in the literature. For example, Qian et al. observed a temporary improvement in the intrinsic dissolution rate for water-insoluble crystalline lurasidone hydrochloride after forming the amorphous form. Moreover, a significant enhancement of dissolution, solubility, and physical stability was observed after forming the co-amorphous form with saccharin [25]. 

The high physical instability of amorphous APIs, with a tendency to convert to crystalline forms, is a limitation in their use. The solution to this problem may be to obtain co-amorphous systems, where APIs inhibit their mutual conversion to crystalline forms [26]. Dengale et al. observed, for ritonavir-indomethacin systems, a nearly 3-fold increase in solubility of amorphous APIs in comparison to crystalline forms [27]. Other examples in which APIs stabilized each other’s amorphous form are the results obtained by Löbmann et al., researchers for indomethacin and naproxen [28], while Singh et al., showed that the combination of glipizide and atorvastatin provided more stability and improved dissolution of both APIs [29].

Another approach is to combine the amorphous API with excipients absorbing humidity as the presence of water can promote the conversion of amorphous forms to crystalline ones, while excipients, by acting as a hindrance, may block the formation of the crystal lattice [30,31,32]. What is more, combining API with excipients may also affect solubility, permeability, and stability and may modify bioavailability [33]. In their study, Ali et al. presented an increase in clozapine solubility and dissolution in the presence of citric, tartaric, and oxalic acids [34]. In turn, Masuda et al. obtained the system comprised of acyclovir and citric acid, which showed improvement of stability and in vitro skin permeation [35]. On the other hand, it was reported that, in combination with saccharine, carvedilol had better stability and solubility [36]. Ana Borrego-Sánchez et al. also studied a drug with low water solubility—praziquantel—for which the dissolution rate is a factor that limits bioavailability. The team obtained praziquantel–clay and praziquantel–calcium carbonate systems, successfully achieving accelerated release and increased solubility for the drug [37,38].

Bearing in mind the specifics of the medical use of vardenafil, its potential new uses, as well as its properties and limitations resulting from them, it was planned to obtain a VAR form with improved physicochemical properties. The stages of our research included: (i) obtaining and confirming the identity of the amorphous form of VAR and preparation of crystalline and amorphous VAR mixtures with excipients, (ii) evaluation of changes in dissolution rate and changes in permeability through biological membranes simulating gastrointestinal epithelium for amorphous form of VAR and its systems with selected excipients, and (iii) assessment of physical stability of amorphous VAR.

## 2. Results

The limitations of VAR use are associated with its physicochemical properties—primarily its low solubility [39]. Especially when using VAR as a drug in erectile dysfunction, a short time of appearance of pharmacological effect is expected. Our approach to improving the physicochemical properties of VAR is based on obtaining its amorphous form stabilized with excipients from the saccharides group with additional functionalities (increasing solubility, masking the taste, and modifying the release of the active substance). In order to assess the impact of physical modification of VAR form, we estimated the changes in physicochemical properties such as dissolution rate and permeability in regards to the crystalline form of the drug. To verify the stability of amorphous VAR, we monitored its chemical and physical stability for one year.

The amorphous VAR was obtained using freeze-drying, and its amorphous character was confirmed by X-ray powder diffraction (PXRD) and differential scanning calorimetry (DSC), supported by FT-IR spectroscopy, coupled with DFT calculations (optimized geometry of VAR is available in Figure S1 in Supplementary Materials).

**Figure 1:**
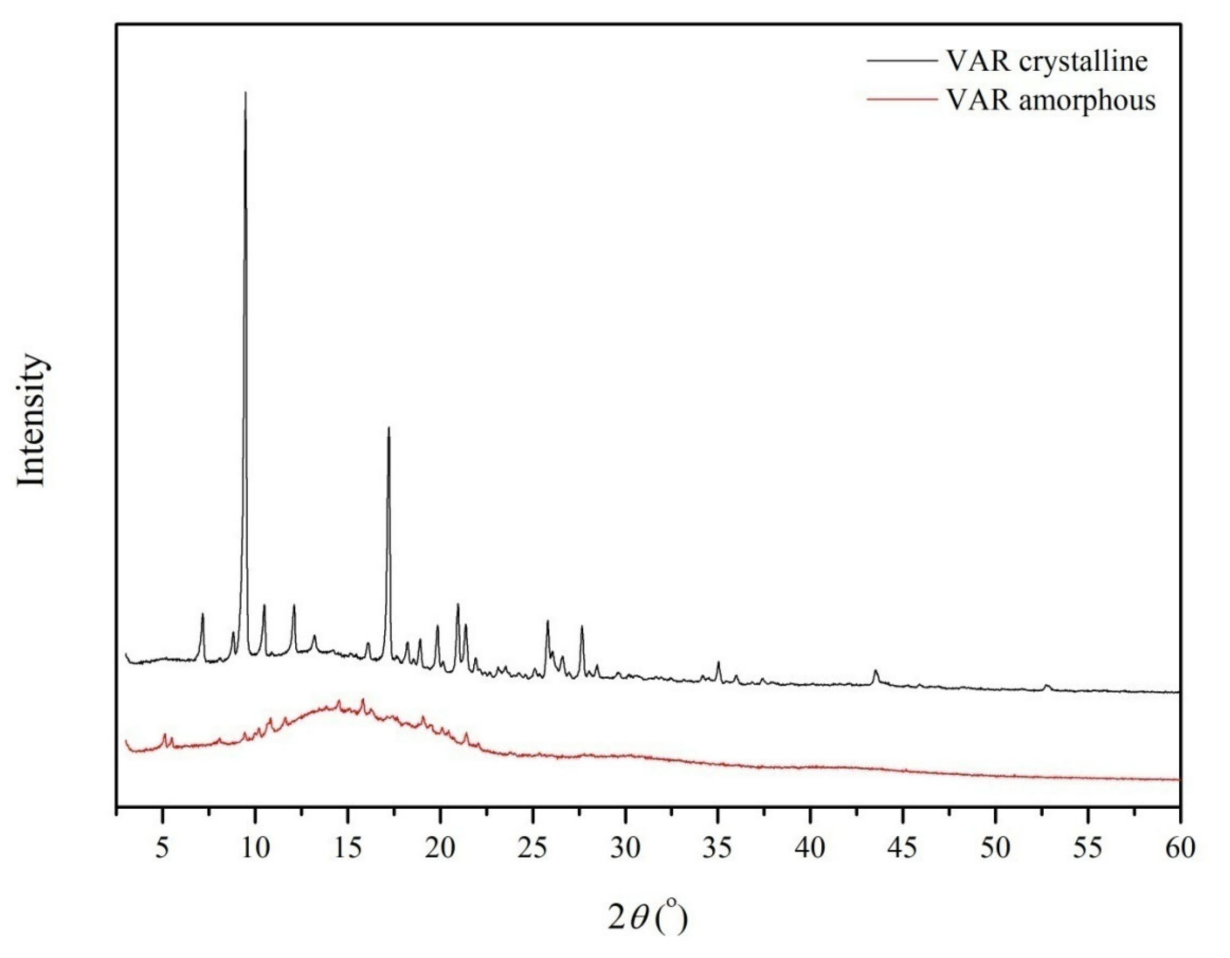
X-ray powder diffraction (PXRD) of crystalline and amorphous forms of vardenafil hydrochloride (VAR).

The PXRD pattern of a crystalline API exhibited sharp peaks, while an amorphous VAR showed a broad background described as a ‘halo’ pattern (Figure 1). The PXRD data confirmed the presence of the crystalline VAR. Meanwhile, on the diffractograms of amorphous VAR dispersion, the disappearance of characteristics for the crystalline form Bragg signals was observed.

**Figure 2:**
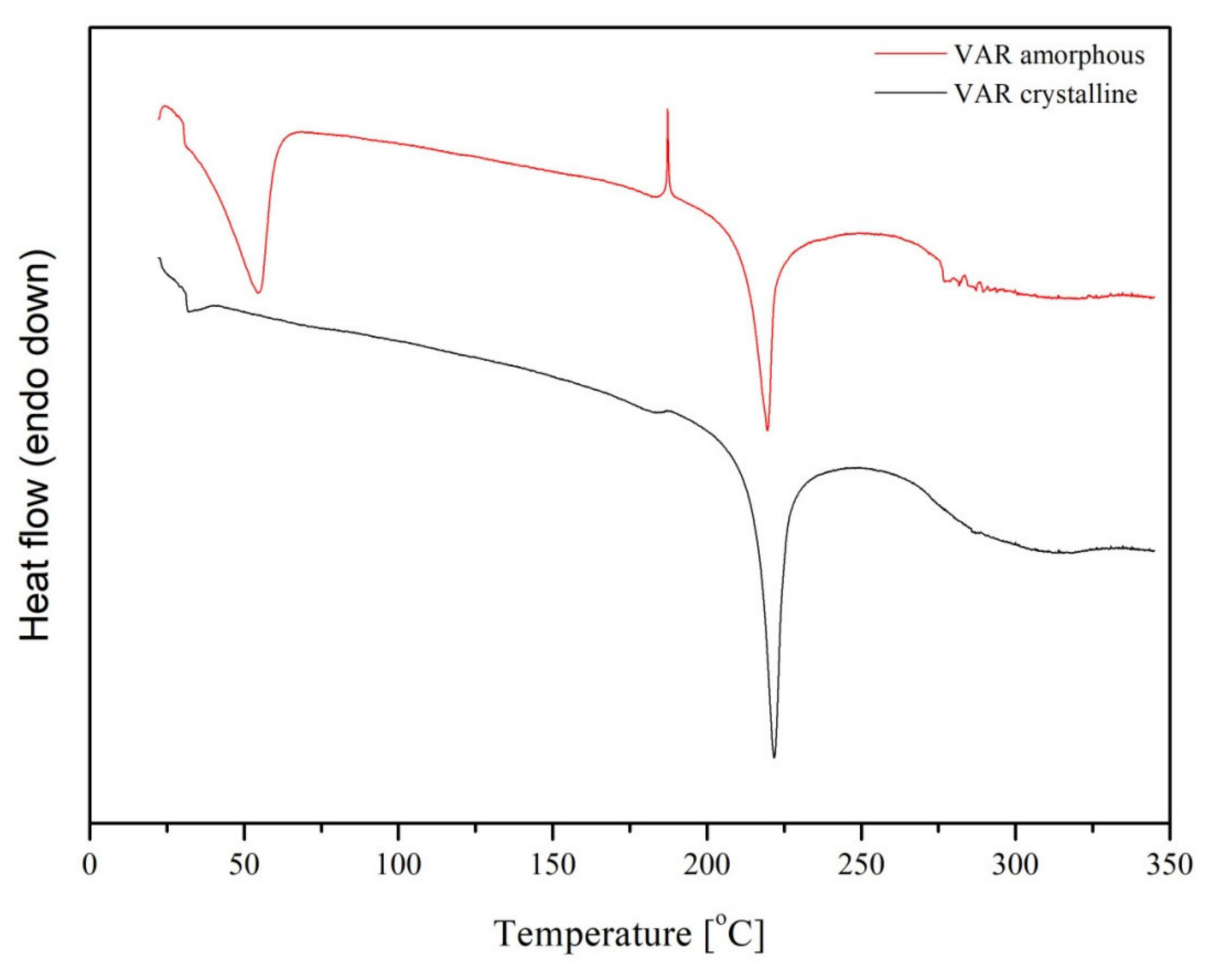
Differential scanning calorimetry (DSC) of crystalline and amorphous VAR.

DSC studies revealed the endothermic peak, which corresponds to the melting point at a temperature of 219 °C in both amorphous and crystalline VAR thermograms. This value is in line with the reported melting temperature of VAR [40]. In DSC results, it is difficult to distinguish glass transition temperature (*T*_g_), which is the characteristic feature of amorphous solids. It may be caused by the appearance of an endothermic peak at about 55 °C, which may blur the view of *T*_g_. However, in the same DSC profile, exothermic peak at the temperature of 185 °C can be noticed, and this can be related to the occurrence of cold crystallization of the sample. On this basis, it can be concluded that at the beginning of the measurement, the sample existed in the amorphous state, but it converted into crystal form during the DSC study. Furthermore, this statement can explain the appearance of the endothermic peak, corresponding to the melting point of VAR that can be seen in the DSC curve for the amorphous sample (Figure 2).

The identification of characteristic bands within the FT–IR spectra of VAR was supported by comparison with theoretical spectra obtained through applying the DFT with the B3LYP hybrid functional and the 6-311G(2d,2p) basis set (Figure S2 and Table S1 in Supplementary Materials).

**Figure 3:**
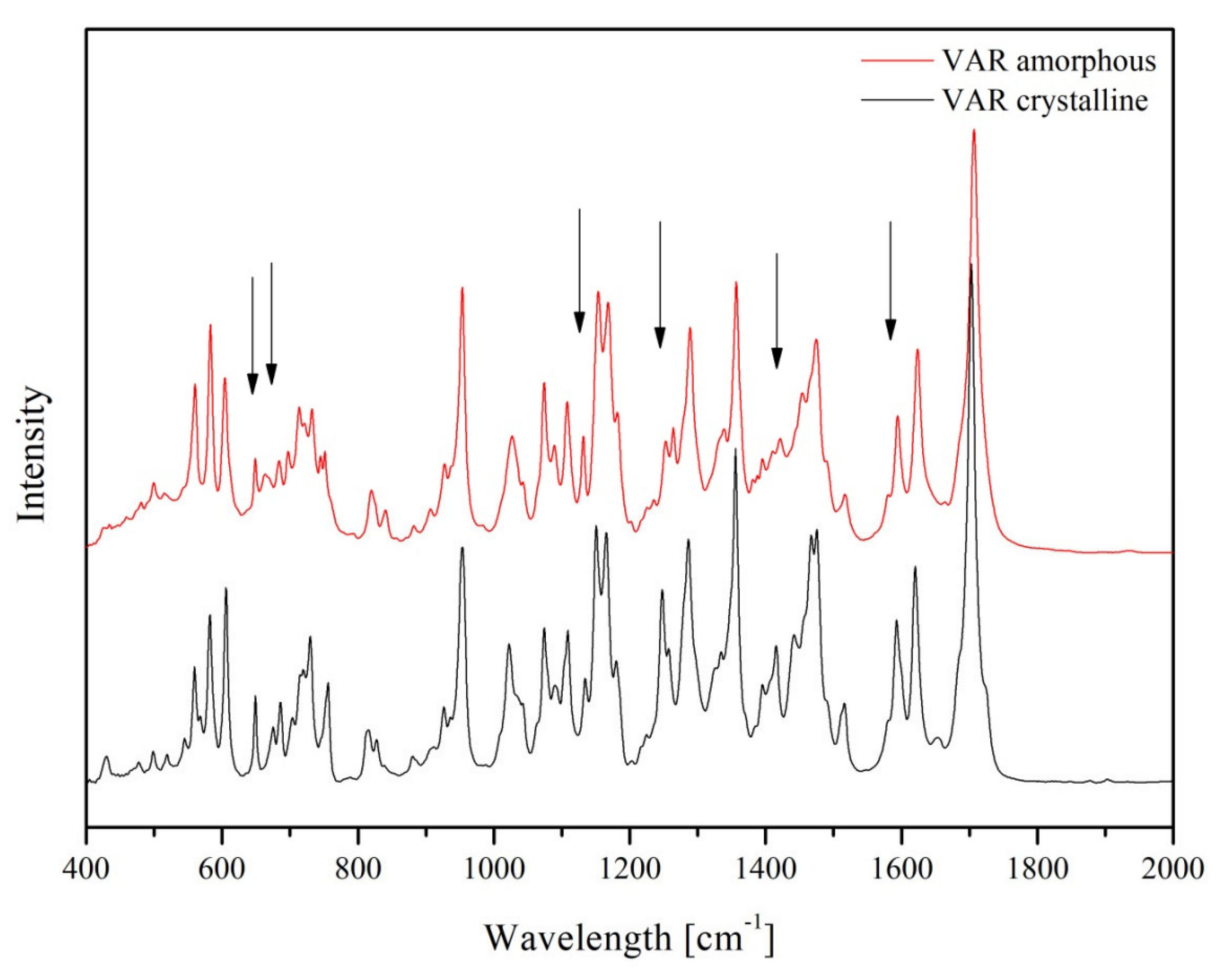
Fourier-transform infrared spectroscopy (FT–IR) of crystalline and amorphous form of VAR.

Comparison of VAR infrared spectrum bands indicates changes in deformation vibrations of C–C bonds in the phenyl ring (at 649 cm^−1^), deformation vibrations of C–H bonds in the pyrazolo[4,3-d]pyrimidin rings (at 689 cm^−1^), stretching vibrations of C–C bonds in the phenyl ring (at 1148 cm^−1^), stretching vibrations of S=O and N–S bonds in the pyrazolo[4,3-d]pyrimidin rings (at 1248 cm^−1^), scissoring vibrations of C–H bonds and rocking vibrations of C–H bonds (at 1442 cm^−1^), and stretching vibrations of C=O bonds (at 1594 cm^−1^) as essential for distinguishing character bands of the amorphous and crystalline form of VAR (Figure 3).

The second part of the research concerned the evaluation of amorphous VAR in regards to apparent solubility and permeability through membranes simulating gastrointestinal epithelium. The UHPLC-DAD method was applied during solubility and permeability studies and the evaluation of the chemical stability of VAR and was validated according to ICH guidelines [41]. As a reference data of apparent solubility, permeability through membranes simulating gastrointestinal epithelium, and chemical stability, values for a crystalline form of VAR were determined.

**Figure 4:**
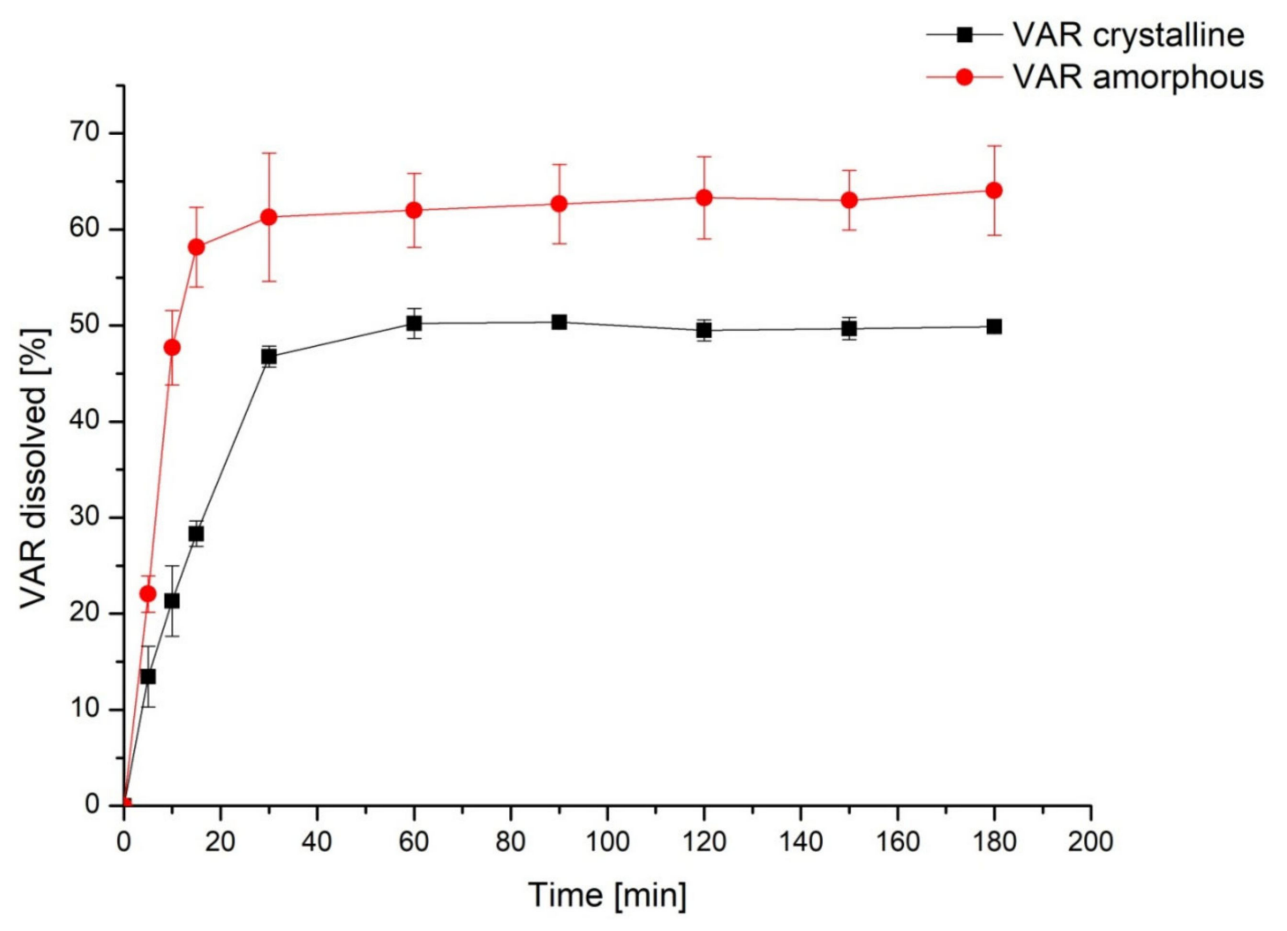
Apparent solubility profiles of crystalline and amorphous forms of VAR.

There is no need for the share of energy needed to break up the crystal lattice dissolving amorphous API. Hence this is a faster process than in the case of crystalline forms. As expected, our results confirmed that the amorphous form of VAR exhibited a nearly 1.3-fold higher amount of drug released (64.06%) than the crystalline one (49.89%) (Figure 4). Figure 4 presents dissolution profiles of crystalline and amorphous forms. The amorphous form improved the total amount of dissolved VAR, and so, on this basis, it can be assumed that it also increased the solubility of the drug with respect to crystalline form.

**Figure 5:**
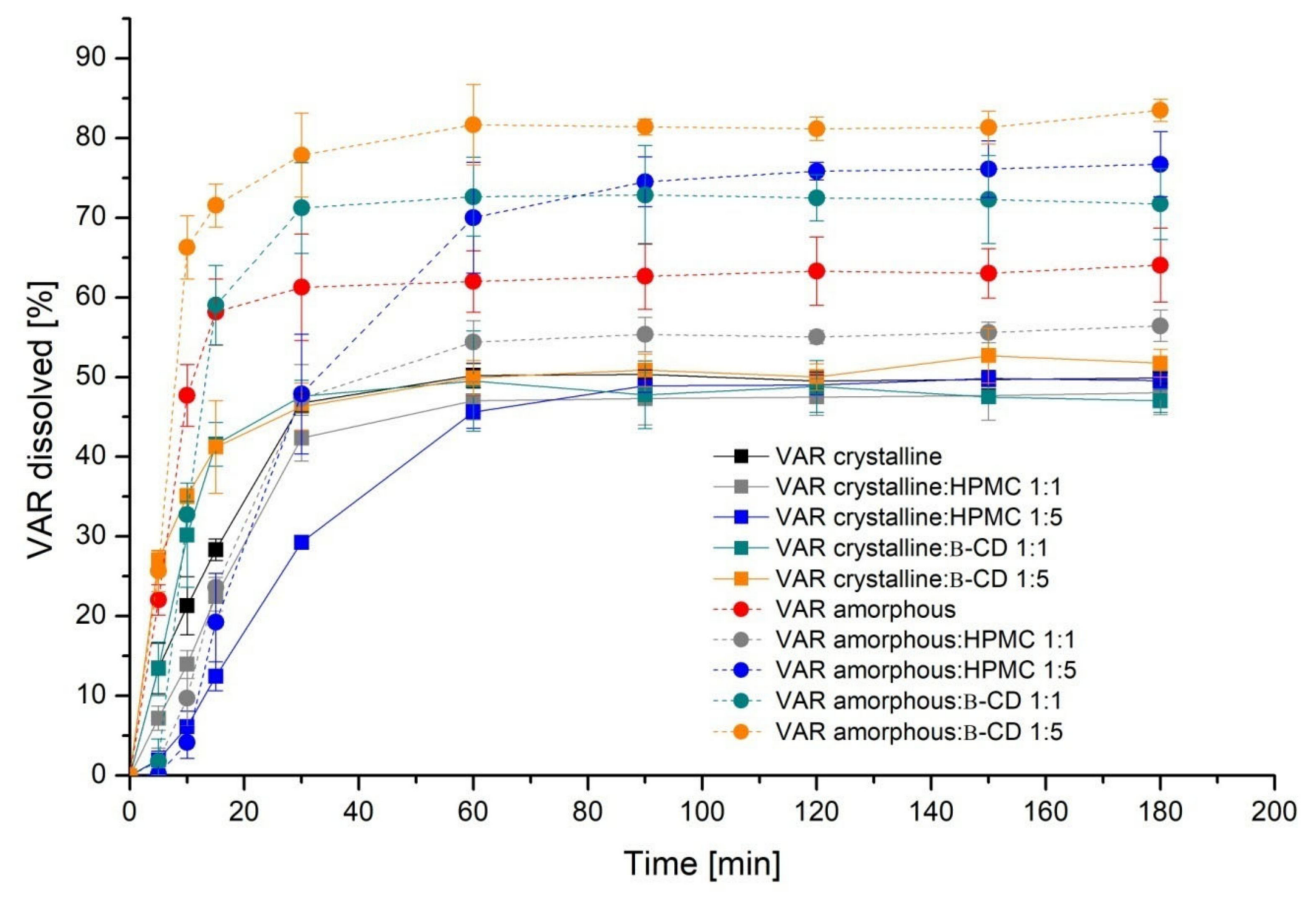
Dissolution profiles of crystalline and amorphous forms of VAR and its systems with excipients (HPMC and β-CD) at 1:1 and 1:5 API:excipient ratio.

The systems of VAR in crystalline and amorphous form with HPMC and β-CD were created in API:excipient ratio of 1:1 and 1:5. Combinations of the amorphous VAR with HPMC and β-CD were characterized by a faster solubility rate of VAR than its amorphous free form. In addition, the amorphous VAR systems with β-CD and HPMC were characterized by an even higher solubility rate of VAR. The ratio of VAR to the excipients used was also important. For 1:5 systems, greater solubility rate improvements were obtained (Figure 5). The system of 1:5 amorphous VAR:β-CD turned out to enhance the dissolution rate the best.

The values of fit factors calculated for comparison of crystalline and amorphous VAR were 40.242 (f_1_) and 16.971 (f_2_). According to the reference values of 50–100 for f_2_ and 0–15 for f_1_, this indicated similarity of profiles—these values indicated the difference between profiles of apparent solubility of amorphous and crystalline VAR forms. For comparing the VAR crystalline profile with its systems and the VAR crystalline systems with excipients, only for VAR crystalline vs. VAR crystalline:HPMC (1:5), the f_1_ value indicated a difference in profiles. Simultaneously, the f_2_ values showed a similarity for the comparison of VAR crystalline:β-CD (1:1) and VAR crystalline:HPMC (1:5), and for the other crystalline VAR systems, they indicated a difference. For the amorphous VAR systems, the difference in profiles was obtained for both the f_1_ and f_2_ coefficients, except for the f_2_ value, for comparisons of amorphous VAR vs.VAR amorphous:β-CD (1:1). The f_1_ and f_2_ factors determined for crystalline and amorphous VAR as well as for other systems are presented in Table 1.

**Figure 6:**
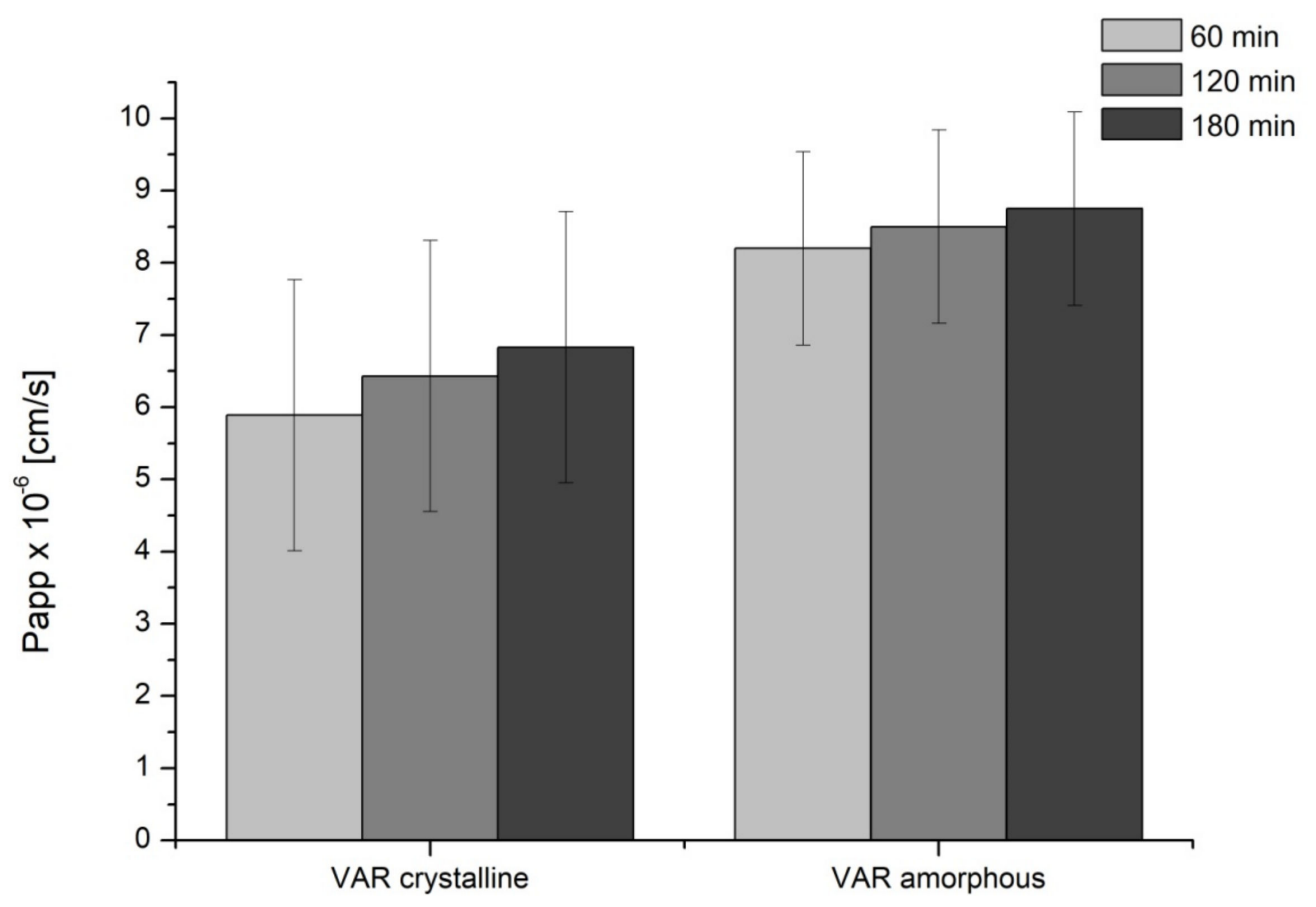
Apparent permeability coefficients (P_app_) of amorphous and crystalline forms of VAR.

As a consequence of changes in the amorphous VAR solubility, the permeability through membranes simulating gastrointestinal epithelium by passive diffusion was evaluated. With the use of the PAMPA gastrointestinal (GIT) model, the values of apparent permeability coefficient (P_app_) were determined for amorphous and crystalline forms of VAR at 60 min, 120 min, and 180 min after the start of the study (Figure 6). As expected, VAR can be classified as a well-permeable drug because the apparent permeability coefficient is more than 6 × 10^−6^ cm/s [42]. Already for the amorphous VAR alone (P_app_ = 8.75 × 10^−6^ cm/s), an increase in permeation was observed at the time points compared to the crystalline form of VAR (P_app_ = 6.83 × 10^−6^ cm/s).

**Figure 7:**
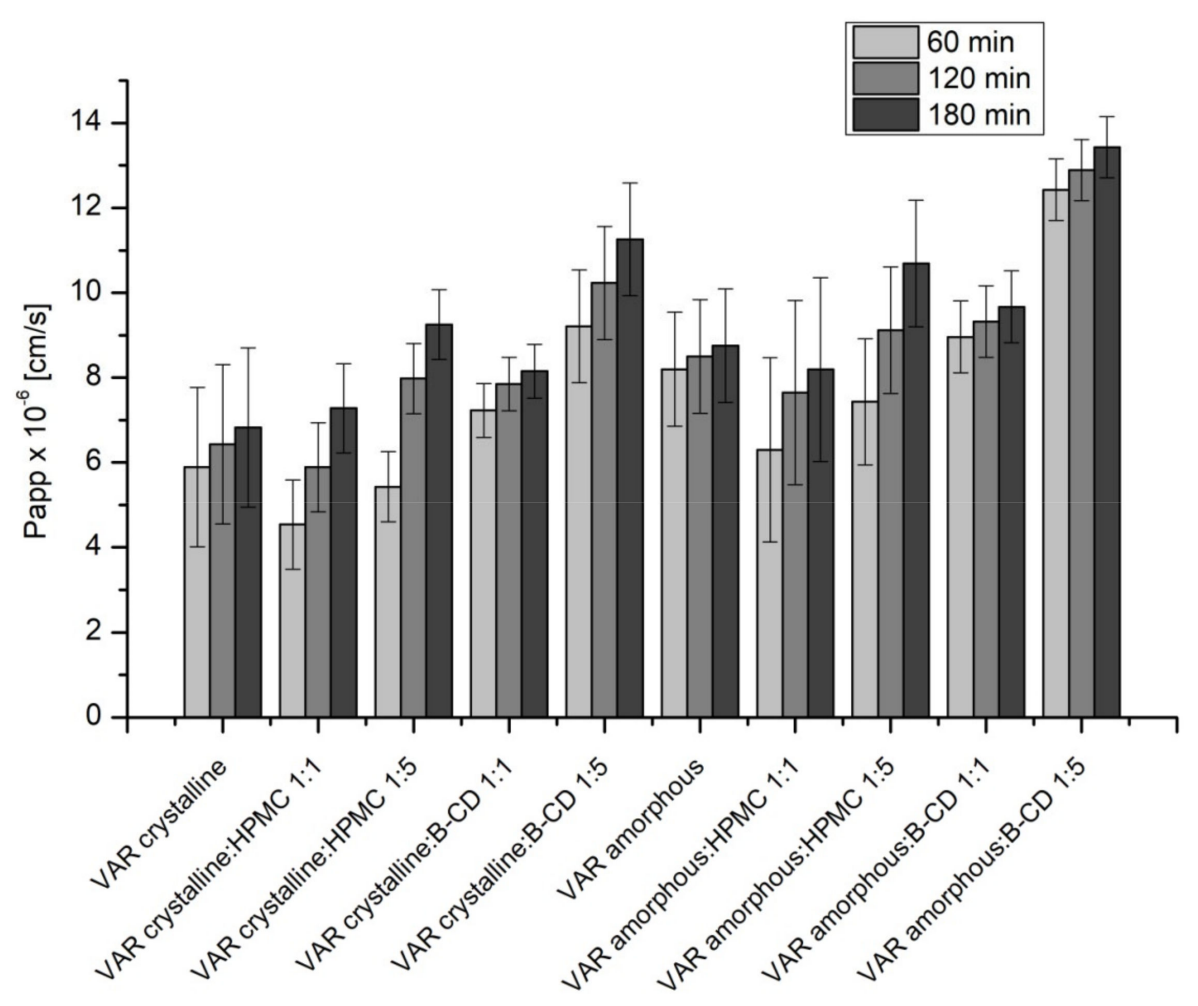
Apparent permeability of amorphous and crystalline of VAR in free form and systems with HPMC and β-CD in 1:1 and 1:5 ratio.

Even higher permeation values were acquired for amorphous VAR in the systems of HPMC and β-CD. The highest values (P_app_ = 13.43 × 10^−6^ cm/s) were obtained for the systems of amorphous VAR with β-CD in the ratio of 1:5 (Figure 7). Furthermore, all amorphous combinations showed improvement of permeability in regards to crystalline free form. However, only the amorphous VAR:β-CD system in the 1:5 ratio exhibited better permeability than the combination of crystalline VAR with β-CD in the 1:5 ratio. When comparing the amorphous system to its crystalline equivalents, it can be concluded that the permeability improved in favor of the amorphous form in all cases. The permeation results using the PAMPA model based on the assessment of concentration changes obtained by passive diffusion correlate with changes in solubility of VAR associated with a change in its combination with selected excipients.

Statistical analysis of the permeability studies results revealed that the P_app_ values obtained for the compositions both amorphous and crystalline VAR with β-CD in the ratio API:excipient 1:5 were statistically significant (*p* ≤ 0,05) (Table S2, Supplementary Materials).

The chemical stability of VAR after the preparation of its amorphous form was tested using the HPLC-DAD method. No change in the peak area and VAR retention time was observed, suggesting its required chemical stability. To assess physical stability, the authors monitored changes in the amorphous VAR using the PXRD technique (Figure S3 in Supplementary Materials). The stability of amorphous VAR was confirmed for over seven months. The first changes in Bragg signals, indicating the formation of a different crystalline form than the original one, appeared after this period.

## 3. Discussion

VAR belongs to the BCS class II, i.e., its bioavailability is limited by poor solubility and amounts to 15% [5]. Such low bioavailability is also related to the deactivation of VAR during the first-pass effect through the liver. Therefore, when designing amorphous VAR systems, the assumption to increase dissolution was adopted. The enhancement of dissolution may provide the acceleration of absorption of VAR, and therefore it contributes to better bioavailability. With improved dissolution rates and permeability, amorphous dispersions could minimize the negative effect of hepatic metabolism by enabling a higher dose of the drug to reach the systemic circulation.

The method which allows the amorphous form of VAR to be obtained was freeze-drying. The applied amorphization technique has many advantages in relation to the possibility of scaling this process with good performance. For other amorphization techniques—vitrification, cryo-milling, or milling with the addition of a solvent—a severe problem is the process scaling e.g., mechanical stress and solvent consumption [43].

It is worth emphasizing that the amorphous form of vardenafil was stable for over six months.

To solubilize the amorphous VAR, its systems with HPMC and β-CD were created in the ratio API:excipient of 1:1 and 1:5. The possibility of solvolysis, taste masking, and modification of the VAR release algorithm was taken into account as additional criteria for the selection of excipients [44,45,46]. Combinations of the amorphous VAR with β-CD were characterized by a faster solubility rate of VAR compared to its amorphous free form. When it comes to VAR:HPMC systems, the one in the 1:5 ratio showed a higher percentage of dissolved VAR than amorphous free form. On the other hand, the combination of VAR and HPMC in the 1:1 ratio exhibited a worse dissolution profile compared to amorphous VAR. The more potent solubilizing properties of β-CD proved to modify the VAR solubility rate strongly. The usage of HPMC was intended to prolong the release of API.

The results obtained in the study performed by authors in terms of the improvement of solubility through amorphization correspond with other researchers’ outcomes. Better solubility of the amorphous form was described by Aucamp et al. for azithromycin obtained by quench cooling of the melt—it was proved to be significantly more soluble in water and buffers of pH 6.8 and 7.2 [47]. Also, Lepek et al. managed to observe better solubility of amorphous telmisartan forms in their study—two amorphous forms of telmisartan, obtained by two different methods (vitrification and cryogrinding), and both exhibited higher solubility than their crystalline form [48].

Improving permeability by solubility enhancement, which was observed during the authors’ study, has been previously proved by scientific reports. In our study, we confirmed the advantageous effect of amorphization on permeability. The amorphous systems showed better permeability than their crystalline forms.

Bearing in mind the observed changes in the systems of amorphous VAR, the authors would like to draw attention to the usefulness of the FT–IR spectroscopy technique in monitoring the state of the tested samples. The usefulness of spectral methods in monitoring the conversion of amorphous API into crystalline forms has already been confirmed for many APIs. The use of the FT–IR technique enabled Craye et al. to characterize the possible interactions between the components of simvastatin–lysine amorphous systems obtained using sodium lauryl sulfate; in addition, changes in these interactions provided data on the stability of the systems [49]. Similarly, Sharma et al. used FT–IR to study changes in the interactions of system components, which reported the stability of the system [50].

In our DSC study, the cold crystallization is visible. This event can be explained by the fact that along with the heating of the sample, there is an increase in molecular mobility of the system, which may further trigger the arrangement of the molecules and formation of the crystal when the activation energy is exceeded. This situation occurred in our investigation of amorphous VAR, and since the analyzed drug was studied without excipient, no obstacle could inhibit the crystal formation by reducing molecular mobility. The appearance of cold crystallization event in the DSC studies of amorphous drugs has also been described in the cases of Sildenafil, Efavirenz, and Biclotymol [51,52,53].

The products on the market contain vardenafil hydrochloride in crystalline form for oral administration or as an orally disintegrating tablet. Considering the results of improved dissolution rate and permeability in vitro obtained for the amorphous form of vardenafil hydrochloride compared to the crystalline form, this may be useful information and the first step for more detailed research into the use of the amorphous form of vardenafil hydrochloride in medicinal products. Taking into account growing interest in the use of VAR in the treatment of diseases other than erectile dysfunction and bearing in mind low bioavailability, the assessment of the impact of combining amorphous VAR with selected saccharides on dissolution rate and permeability can be the first stage, when it comes to the development of novel formulations containing VAR in amorphous form. As an additional advantage of obtaining amorphous VAR, financial benefits should be mentioned. The use of amorphous VAR will allow its bioavailability to be increased and, consequently, this will reduce the use of the therapeutic dose to obtain the required pharmacological effect.

## 4. Materials and Methods

### 4.1. Materials

Vardenafil hydrochloride (purity > 98%) was supplied by Polpharma (Starogard Gdański, Poland), while excipients—hydroxypropyl methylcellulose (viscosity 6 cP; average Mn~10000; 29 wt. % methoxy, 7 wt. % propylene oxide) and β-cyclodextrin were obtained from Sigma Aldrich Chemie (Schnelldorf, Germany). Acetonitrile of super purity was supplied by Romil (Waterbeach, England) and formic acid (100%) by Avantor Performance Materials (Gliwice, Poland). High-quality pure water was prepared using USFT-801 water purification system (Millipore, Burlington, VT, USA) and the Elix SA 67120 millipore purification system (Molsheim, France). Hydrochloric acid, sodium chloride, and all other chemicals were obtained from Avantor Performance Materials (Giwice, Poland). For permeability studies Prisma HT, GIT lipid solution, Acceptor Sink Buffer supplied by Pion Inc. (Billerica, MA, USA) were used.

### 4.2. Preparation of the Amorphous VAR

VAR was converted to amorphous forms in the freeze-dryer, ScanvacCoolSafe 55-4 Basic. The preparation of solutions for lyophilization involved weighting the API and dissolving it in water, using 1 part of the API to 10 parts of the solvent ratio. The concentration of obtained solutions was 0.1 mg/mL. These solutions were stirred on a magnetic stirrer for 5 h to ensure complete dissolution of the drug. Then the solution was divided and poured into round-bottom flasks and was frozen overnight. Flasks with frozen solutions were placed in the manifolds of the freeze-dryer. The conditions of the freeze-drying process were −55 °C, 6 hPa, and the process lasted for 72 h. The total amount of VAR used for the preparation of amorphous form was 700 mg and the obtained quantity was 650 mg of amorphous VAR.

### 4.3. Preparation of the Physical Mixtures of Crystalline or Amorphous VAR with Excipients

Solid dispersions of crystalline and amorphous VAR forms with excipients were prepared using hydroxypropyl methylcellulose and β-cyclodextrin (API:excipient weight ratio 1:1 and 1:5 for each system) by grinding in a mortar and then stored in a desiccator at ambient temperature. About 80 mg of amorphous VAR was used to prepare each system.

### 4.4. Identification of Amorphous VAR

The identity of amorphous VAR was conducted using X-ray powder diffraction studies (PXRD), differential scanning calorimetry (DSC), and FT–IR spectroscopy coupled with DFT calculations as a support method.

#### 4.4.1. X-ray Powder Diffraction (PXRD)

For analysis of VAR, the X-ray powder diffraction (PXRD) technique was used. Obtained results were collected by PANalitycal Empyrean system with Cu lamp (1.54056 Å) (Malvern Panalytical Ltd., Poznan, Poland). The measurements were performed at the scanning range 3°–50° at 5*θ* range with a step size of 0.017° and a step time of 15 s/step; the source parameters were 45 kV and 40 mA.

#### 4.4.2. Different Scanning Calorimetry (DSC)

DSC analyses of amorphous and crystalline forms of VAR were performed using a differential scanning calorimeter (DSC 204 F1 Phoenix, NETZSCH-Gerätebau GmbH, Selb, Germany). The cell constant calibration method was applied to analyze the DSC patterns of the samples from 25 °C to 350 °C at a heating step 0.08 °C, under a constant flow of 10 mL/min of nitrogen atmosphere.

#### 4.4.3. FT–IR Spectroscopy

Spectra of crystalline and amorphous forms of VAR were obtained with IR grade KBr in the ratio of 1:100, and corresponding pellets with excipients were prepared in the hydraulic press by applying 8-metric-ton of pressure. The spectra were recorded at the range 400 and 4000 cm^−1^, with an FT–IR Bruker Equinox 55 spectrometer (Bruker, Bermen, Germany) equipped with a Bruker Hyperion 1000 microscope (Bruker, Bermen, Germany). Quantum chemical calculations based on DFT were carried out to study changes in positions and intensity in experimental FT–IR spectra for studied forms of VAR. The calculations were made using the Gaussian 03 package (Wallingford, CT, USA).

### 4.5. Studies of Apparent Solubility and Permeability

The influence of amorphous and crystalline VAR forms on the apparent solubility, permeability through an artificial membrane simulating gastrointestinal permeation, and chemical stability was carried out using chromatographic determinations. For evaluation of the compatibility between VAR and excipients, chemical stability studies were added. In those studies, the changes in VAR concentrations were determined using ultra-high-performance liquid chromatography with the diode array detector (UHPLC-DAD) method. The UHPLC system (Dionex Thermoline Fisher Scientific, Waltham, MA, USA) was equipped with a high-pressure pump (UltiMate 3000), an autosampler (UltiMate 3000), and a DAD detector (UltiMate 3000). For data processing and acquisition, Chromeleon software version 7.0 from Dionex Thermoline Fisher Scientific (Waltham, MA, USA) was used. The determination was achieved on a Kinetex C18 2.6 µm (100 × 2.10 mm) column, at the temperature of 30 °C, using a mobile phase composed of 0.1% formic acid:acetonitrile (70:30 *v*/*v*) at a flow rate of 0.25 mL/min. The injection volume was 1.0 µL, and the wavelength of detection was 250nm. The duration time of UHPLC analysis was 4 min and the retention time of VAR was approximately 1.2 min.

#### 4.5.1. Apparent Solubility

The apparent solubility of amorphous and crystalline VAR forms and their combinations with excipients—hydroxypropyl methylcellulose and β-cyclodextrin—were performed using an Agilent 708-DS Dissolution Apparatus (Agilent, Santa Clara, CA, USA) in non-sink conditions. The standard paddle apparatus was used at 37 ± 0.5 °C of the medium with a stirring speed of 50 rpm. The amorphous and crystalline forms of VAR and their mixtures with excipients (API:excipient weight ratio: 1:1 and 1:5) were weighed into gelatin capsules, corresponding to 20 mg of pure drug, and placed in 500 mL of simulated gastric acid of pH 1.2. To prevent the capsules from flotation on the surface of the medium, they were placed into sinkers. During the study, the samples of 5.0 mL of the solution were collected at specified time intervals, then replaced with 5.0 mL of the pure medium of 37 ± 0.5 °C. The samples were filtered through a 0.2 μm membrane filter (Acrodisc^®^ Syringe Filter 0.2 µm, 13 mm, Pall Corporation, New York, NY, USA) and analyzed using chromatographic determinations. The three replicates for each sample were assayed. The similarity of dissolution profiles of amorphous and crystalline forms of VAR was established on the *f*_1_—difference parameter (Equation (1)) and *f*_2_—similarity parameter (Equation (2)) and was defined by the following equations:

(1) $$f_{1}=\frac{\sum_{j=1}^{n}\left|R_{j}-T_{j}\right|}{\sum_{j=1}^{n}R_{j}}\times 100$$

(2) $$f_{2}=50\times \log\left({\left(1+\left(\frac{1}{n}\right)\overset{n}{\underset{j=1}{\sum }}{\left|R_{j}-T_{j}\right|}^{2}\right)}^{-\frac{1}{2}}\times 100\right)$$

where *n* is the number of withdrawal points, *R_j_* is the percentage dissolved of reference sample at the time point *t*, and *T_j_* is the percentage dissolved of the test sample at the time point *t*. According to the Moore and Flanner model, for the *f*_2_ value, 100 suggests that test and reference profiles are identical, values between 50 and 100 indicate their similarity, and values <50 imply an increase in differences between release profiles. For *f*_1_ values of 0–15, profiles are considered similar (value 0 suggests identical profiles), while values >15 show profile dissimilarity [54].

#### 4.5.2. Permeability Studies

Permeability of amorphous and crystalline VAR forms in free forms as well as in combinations with selected excipients through artificial biological membranes was conducted using a parallel artificial membrane permeability assay (PAMPA) system from Pion, Inc. It consists of two matching 96-well plates called a donor (bottom plate) and an acceptor (top plate), separated by a 120-μm-thick microfilter disc, which, for this study, was coated with a 20% (*w*/*v*) dodecane solution of a lecithin mixture (Pion Inc., Billerica, MA, USA). The donor buffer was adjusted to an acidic pH value. The testing samples of amorphous and crystalline VAR and their mixtures with excipients were prepared by dissolving in water, prepared in a different 96-well filter plate, and these were added to the donor compartments. The donor and acceptor plates were sandwiched together and incubated at 37 °C for 3 h in a humidity-saturated atmosphere. At specific time intervals, plates were separated in order to investigate VAR concentrations using the HPLC-DAD method. The apparent permeability coefficient (*P_app_*) was calculated according to Equation (3).

(3) $$P_{app}=\frac{-ln\left(1-\frac{C_{A}}{C_{equilibrium}}\right)}{S\times \left(\frac{1}{V_{D}}+\frac{1}{V_{A}}\right)\times t}$$

where *V_D_* is the donor volume, *V_A_* is the acceptor volume, *C_equilibrium_* is the equilibrium concentration $C_{equilibrium}=\frac{C_{D}\times V_{D}+C_{A}\times V_{A}}{V_{D}+V_{A}}$, *S* is the membrane area, and *t* is the incubation time (in seconds).

Compounds with a *P_app_* < 1 × 10^−6^ cm/s are classified as low permeability and compounds with a *P_app_* > 1 × 10^−6^ cm/s are classified as ones with high permeability [42].

### 4.6. Studies of Chemical and Physical Stability

#### 4.6.1. Chemical Stability Studies

For the accelerated aging test, samples of amorphous and crystalline forms of VAR with selected excipients (1:1 and 1:5 ratio) were weighed into 5 mL vials. To evaluate their stability in increased air humidity, they were placed in heat chambers at 60 °C in a desiccator containing a saturated solution of inorganic salt—sodium chloride (~76.4% relative humidity (RH)). The vials were removed at specified time intervals, then cooled to room temperature, and the contents were dissolved in water and injected into the chromatographic column. The period of conduction of the accelerated aging test was 7 weeks.

#### 4.6.2. Physical Stability Studies

The X-ray powder diffraction (PXRD) technique was used to study the physical stability of the amorphous form of VAR. Samples were tested after being obtained and during the storage in accelerated aging test conditions (60 °C, RH ~ 76.4%) for 7 and 12 months when samples were stored in ambient conditions (room temperature, RH uncontrolled).

### 4.7. Statistical Analysis

The results were analyzed using Statistica software (version 13.0, Stat Soft Inc., Tulsa, OK, USA). The ANOVA test was used to compare the results of the measurements in the apparent permeability studies. The differences were considered to be significant when *p* ≤ 0.05.

## 5. Conclusions

Freeze-drying turned out to be an effective way of obtaining amorphous VAR hydrochloride and did not generate new impurities. The amorphous dispersion of VAR was characterized by a faster dissolution rate and increased penetration dynamics through membranes simulating the walls of the gastrointestinal system. In addition, amorphous VAR combined with β-cyclodextrin (1:5) showed a particular improvement in these two important parameters (dissolution rate and membrane permeation conditioned by passive diffusion) to increase the bioavailability of VAR. β-cyclodextrin also acts as a long-term stabilizer for the amorphous VAR. The results of improving the dissolution rate and permeability obtained for the amorphous form of VAR can be considered an early stage of research for its potential use in medical products. Further research into mixtures of amorphous VAR with excipients or amorphous solid dispersion is required to draw some more detailed conclusions on the subject of amorphous VAR and its stable formulation.

## Figures and Tables

**Table 1:** The values of f_1_ and f_2_ for tested systems. Values indicating the difference of the dissolution profiles are red. Values in green indicate their similarity.

Compared Systems		f_1_	f_2_
VAR_crystalline_	VAR_crystalline_:HPMC (1:1)	10.02	47.09
VAR_crystalline_	VAR_crystalline_:HPMC (1:5)	18.64	33.75
VAR_crystalline_	VAR_crystalline_:β-CD (1:1)	8.87	67.28
VAR_crystalline_	VAR_crystalline_:β-CD (1:5)	13.06	42.06
VAR_crystalline_:HPMC (1:1)	VAR_crystalline_:HPMC (1:5)	13.69	50.49
VAR_crystalline_:β-CD (1:1)	VAR_crystalline_:β-CD (1:5)	9.32	49.93
VAR_crystalline_	VAR_amorphous_	40.24	16.97
VAR_amorphous_	VAR_amorphous_:HPMC (1:1)	28.75	16.93
VAR_amorphous_	VAR_amorphous_:HPMC (1:5)	34.88	36.21
VAR_amorphous_	VAR_amorphous_:β-CD (1:1)	18.43	57.10
VAR_amorphous_	VAR_amorphous_:β-CD (1:5)	28.99	16.74
VAR_amorphous_:HPMC (1:1)	VAR_amorphous_:HPMC (1:5)	30.20	28.42
VAR_amorphous_:β-CD (1:1)	VAR_amorphous_:β-CD (1:5)	23.47	20.38

## Data Availability

The data presented in this study are available in the main body text and the Supplementary Material of the current article.

## Supplementary Materials

The following are available online at https://www.mdpi.com/article/10.3390/ph14050453/s1: Figure S1: Optimized geometry of VAR. DFT method with B3LYP hybrid functional and 6-31G(d,p) basis set, Figure S2: The calculated (blue) and experimental FT-IR spectra of VAR in amorphous (red) and crystalline (black) form, Table S1: Selected experimental and theoretical modes characteristic vibronic features of VAR in crystalline and amorphous form. All number values expressed in cm−1; abbreviations: s, stretching; b, bending; r, rocking; w, wagging; sc, scissoring. Figure S3. PXRD of VAR during physical stability studies.
