# Supplementary material for: Combinations of Freeze-Dried Amorphous Vardenafil Hydrochloride with Saccharides as a Way to Enhance Dissolution Rate and Permeability
Source: Pharmaceuticals (Basel). 2021 May 11;14(5):453. doi: 10.3390/ph14050453 (PMC8151567; doi:10.3390/ph14050453)
Supplement: Supplementary file 1 [file pharmaceuticals-14-00453-s001.zip › pharmaceuticals-1191144-supplementary.pdf]

Supplementary materials: Combination of freeze-dried amorphous vardenafil hydrochloride with saccharides as a way to enhance dissolution rate and permeability

Gabriela Wiergowska, Dominika Ludowicz, Kamil Wdowiak, Andrzej Miklaszewski, Kornelia Lewandowska and Judyta Cielecka-Piontek

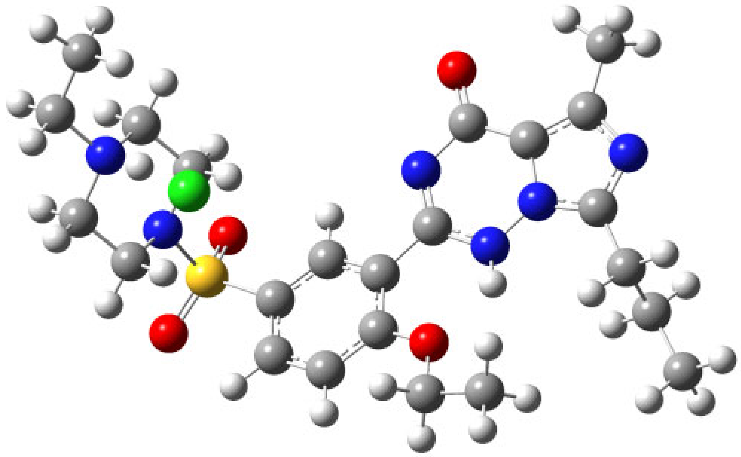

Figure S1. Optimized geometry of VAR. DFT method with B3LYP hybrid functional and 6-31G(d,p) basis set.

The quantum chemical calculations of the normal modes of vibrations enabled the assignment of the bands observed in the experimental spectra to specific vibrations of various functional groups of the molecule. The experimental results were compared with calculations obtained using a basis set (6-31G(d,p)) (FigureS2).

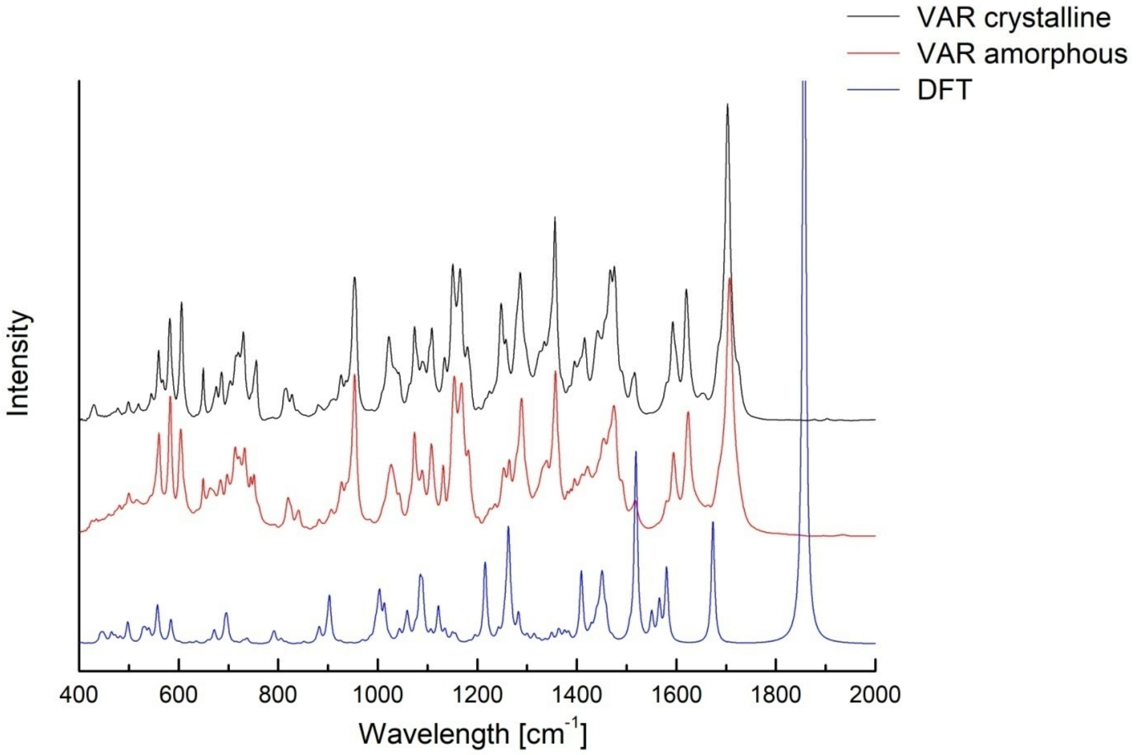

Figure S2. The calculated (blue) and experimental FT-IR spectra of VAR in amorphous (red) and crystalline (black) form.

Table 1. On the basis of the two spectra, we identified and assigned the most important bands observed in Table 1. Selected experimental and theoretical modes characteristic vibronic features of VAR in crystalline and amorphous form. All number values expressed in cm^{-1}; abbreviations: s – stretching, b – bending, r – rocking, w – wagging, sc – scissoring.

Calculation wavelength	Experimental wavelength		Band assignment
	amorphous	crystalline	
465	481	477	def. all molecule
540	560	557	def. all molecule
556	583	581	def. all molecule
584	604	604	C-H w + O=S=O sc
621			def. pyrazolo-pyrimidine ring
635	648	649	def. phenyl ring
671	684	687	def. pyrazolo-pyrimidine ring + C-N w in pyrazolo-pyrimidine ring
695	751	755	N-S s + rocking pyrazolo-pyrimidine ring + def. ethyl-piperazine group
790	820	815	C-H w + C-H t in ethoxy group
806	841	840	C-H t + C-H w in ethyl-piperazine group
851	882	879	C-C s + C-H w in propyl group
883	927	927	piperazine ring
903	954	953	C-N s + C-C s in ethyl-piperazine group
1003	1026	1023	C-C s in propyl group + C-N s in pyrazolo-pyrimidine ring
1016	1043	1041	C-C s in ethoxy group
1058	1074	1075	O=S s + C-H r in ethyl-piperazine group and phenyl ring
1063	1089	1090	O=S s + C-H r in ethyl-piperazine group and phenyl ring
1089	1108	1108	C-H w in ethoxy group and phenyl ring + O=S s
1106	1132	1134	C-N s in ethyl-piperazine group
1121	1153	1148	C-C s in phenyl ring + C-N s in pyrazolo-pyrimidine ring + C-H sc in phenyl ring + def. pyrazolo-pyrimidine ring
1135	1168	1164	C-H w in phenyl ring
1195			C-H t in propyl group
1214	1236	-	C-O s between ethoxy group and phenyl ring
1261	1253	1248	O=S s + C-N s in pyrazolo-pyrimidine ring + C-H r
1282	1264	1257	C-H t in ethyl- piperazine group
1293	1289	1286	C-C s in phenyl ring
1323			C-N s in pyrazolo-pyrimidine ring
1365	1357	1354	C-H sc
1382			C-C s in phenyl ring + N-H r in pyrazolo-pyrimidine ring
1409	1393	1393	N-N s in pyrazolo-pyrimidine ring
1427	1421	1414	C-H sc + C-H r
1442	1454	1442	C-H sc + C-H r
1450	1475	1476	C-H sc + C-H r
1517	1517	1514	C=N s + C-N-H b in pyrazolo-pyrimidine ring
1566			C=C s in phenyl ring + C=N s in pyrazolo-pyrimidine ring
1672	1594	1594	C=O s
1672	1623	1618	C=O s
1858	1707	1702	H-Cl s
2921	2621	2508	C-H s in propyl ring
2934	2680	2570	C-H s in CH3 group in pyrazolo-pyrimidine ring
2952	2722	2668	C-H s in ethyl-piperazine group

2987	2934	2927	C-H s in propyl ring
3006	2967	2964	C-H s in ethyl-piperazine group
3118			C-H s in phenyl ring
3413	3332	3335	N-H s in pyrazolo-pyrimidine ring

Our research dealt with crystalline and amorphous forms of VAR and the changes observed in the spectra confirm that. The most intense bands observed in the FT-IR spectrum of crystalline form are located at 953, 1023, 1075, 1108, 1148, 1164, 1248, 1286, 1354, 1476, 1594, 1618, and 1702 cm^{-1}. In this group, we observed bands related to the most characteristic vibration in this molecule. For example, the band at 1248 and 1594/1618 cm^{-1}correspond to the stretching vibration of the O=S, C-N and C=O bonds of the pyrazine-pyrimidine group, respectively. At lower frequencies (1075cm^{-1}) the band related to the stretching vibration of the O=S bond is also observed, but it has additional components related to the rocking vibration of the C-H bonds in the ethyl-piperazine group and phenyl ring. The band located at 953 cm^{-1} is associated with the C-N and C-C stretching vibration bond in this same group. In contrast, bands related to the stretching vibration of the C-C bond in the propyl group and phenyl ring are located at 1023 and 1148 cm^{-1}, respectively. The other four bands located at 1108, 1164, 1354, and 1476 cm^{-1} are mainly corresponding to the wagging and scissoring vibration of the C-H bonds. The band at 1702 cm^{-1} is related to the stretching vibration of the H-Cl bond. In the absorption IR spectrum, quite strong are the bands about 550-800 cm^{-1}. The characteristic is the band at 755 cm^{-1} related to the stretching vibration of the N-S bond and the band at 604 cm^{-1} that is related to the scissoring vibration of the O=S=O bond. The other bands in this range are primarily related to the deformation of the molecule. The bands above 2500 cm^{-1} correspond to the stretching vibration of the C-H bonds, and the band at 3335 cm^{-1} is related to the stretching vibration of the N-H bond.

When we compared the spectra for crystalline and amorphous forms of VAR, we can see that some of the bands for the amorphous form almost disappear. For example, the band at 1236 cm^{-1}related to the wagging vibration of the C-H bonds in the ethoxy group. The changes in the spectrum are also visible in the range 650 – 850 $cm^{-1,}$ where are located, especially the bands related to the deformation of the molecule and vibration of the C-H bonds. The big changes are observed in the range above 2300 cm^{-1}, where the bands associated with the stretching vibration of the C-H bonds are located. In this region, massive shifts of the bands and changing the shapes of the bands can be seen. The changes in the intensity of the bands are also observed for the bands at 1134, 1248, and 1414 cm^{-1}. These bands are related to the stretching vibration of the C-N bond in the ethyl-piperazine group, stretching vibration of the O=S bond, and scissoring vibration of the C-H bonds. The changes we observe suggest that a smaller distance separates the molecules in the amorphous form, and they are closer to each other. Therefore some bands disappear – especially the bands associated with bond vibration, which are not bound by the ring and, due to their peripheral position in the particle, have more space of movement.

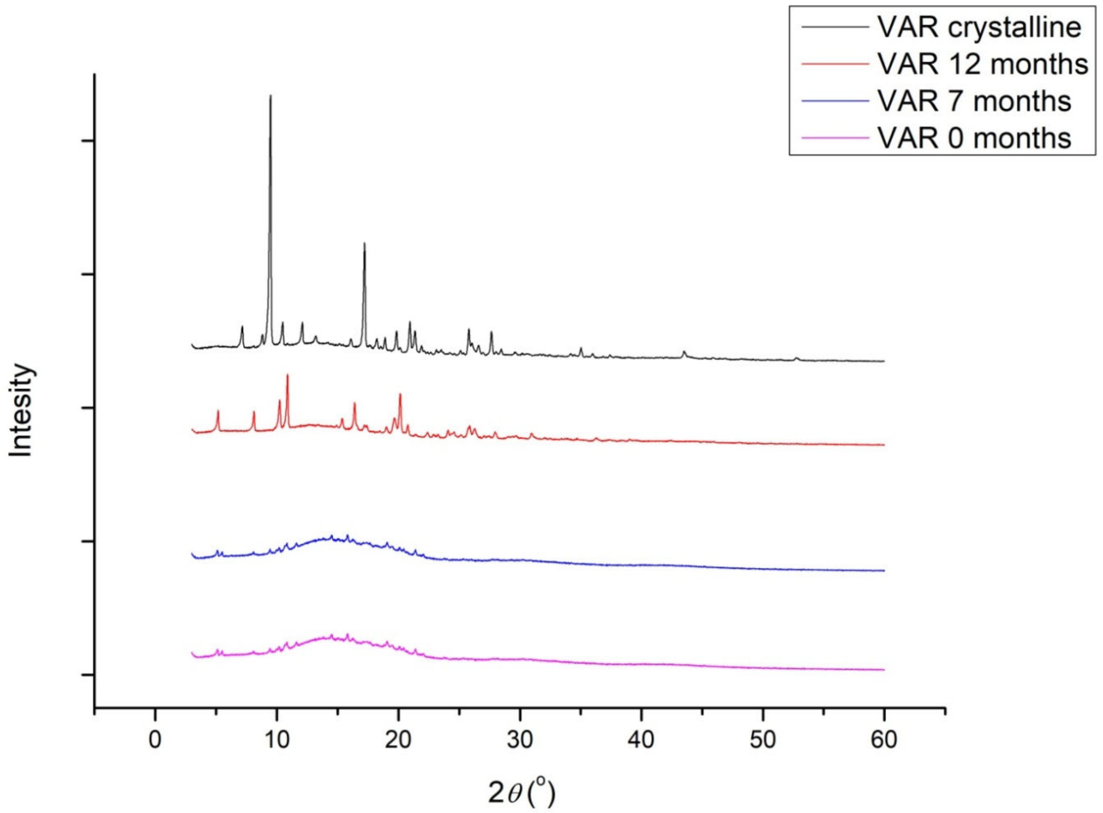

Figure S3. PXRD of VAR during physical stability studies.

Table S2. The results of statistical analysis.

Compared systems		p
**VAR$_{crystalline}$**	VAR$_{crystalline}$:HPMC (1:1)	0.728
VAR$_{crystalline}$	VAR$_{crystalline}$:HPMC (1:5)	0.104
VAR$_{crystalline}$	VAR$_{crystalline}$:β-CD (1:1)	0.300
VAR$_{crystalline}$	VAR$_{crystalline}$:β-CD (1:5)	0.027
VAR$_{crystalline}$	VAR$_{amorphous}$	0.212
VAR$_{amorphous}$	VAR$_{amorphous}$:HPMC (1:1)	0.724
VAR$_{amorphous}$	VAR$_{amorphous}$:HPMC (1:5)	0.175
VAR$_{amorphous}$	VAR$_{amorphous}$:β-CD (1:1)	0.368
VAR$_{amorphous}$	VAR$_{amorphous}$:β-CD (1:5)	0.006
